# A Eukaryotic-Acquired Gene by a Biotrophic Phytopathogen Allows Prolonged Survival on the Host by Counteracting the Shut-Down of Plant Photosynthesis

**DOI:** 10.1371/journal.pone.0008950

**Published:** 2010-01-28

**Authors:** Betiana S. Garavaglia, Ludivine Thomas, Natalia Gottig, Germán Dunger, Cecilia G. Garofalo, Lucas D. Daurelio, Bongani Ndimba, Elena G. Orellano, Chris Gehring, Jorgelina Ottado

**Affiliations:** 1 Molecular Biology Division, Instituto de Biología Molecular y Celular de Rosario, Consejo Nacional de Investigaciones Científicas y Técnicas, Facultad de Ciencias Bioquímicas y Farmacéuticas, Universidad Nacional de Rosario, Rosario, Argentina; 2 Consejo de Investigaciones, Universidad Nacional de Rosario, Rosario, Argentina; 3 Department of Biotechnology, University of the Western Cape, Bellville, South Africa; 4 Computational Bioscience Research Centre, King Abdullah University of Science and Technology, Thuwal, Kingdom of Saudi Arabia; Massachusetts General Hospital, United States of America

## Abstract

*Xanthomonas citri* pv. *citri*, the bacteria responsible for citrus canker posses a biological active plant natriuretic peptide (PNP)-like protein, not present in any other bacteria. PNPs are a class of extracellular, systemically mobile peptides that elicit a number of plant responses important in homeostasis and growth. Previously, we showed that a *Xanthomonas citri* pv. *citri* mutant lacking the PNP-like protein XacPNP produced more necrotic lesions in citrus leaves than wild type infections and suggested a role for XacPNP in the regulation of host homeostasis. Here we have analyzed the proteome modifications observed in citrus leaves infected with the wild type and *XacPNP* deletion mutant bacteria. While both of them cause down-regulation of enzymes related to photosynthesis as well as chloroplastic ribosomal proteins, proteins related to defense responses are up-regulated. However, leaves infiltrated with the *XacPNP* deletion mutant show a more pronounced decrease in photosynthetic proteins while no reduction in defense related proteins as compared to the wild-type pathogen. This suggests that XacPNP serves the pathogen to maintain host photosynthetic efficiency during pathogenesis. The results from the proteomics analyses are consistent with our chlorophyll fluorescence data and transcript analyses of defense genes that show a more marked reduction in photosynthesis in the mutant but no difference in the induction of genes diagnostic for biotic-stress responses. We therefore conclude that XacPNP counteracts the shut-down of host photosynthesis during infection and in that way maintains the tissue in better conditions, suggesting that the pathogen has adapted a host gene to modify its natural host and render it a better reservoir for prolonged bacterial survival and thus for further colonization.

## Introduction

Citrus canker, caused by *Xanthomonas citri* pv. *citri* (Xcc), is one of the most devastating diseases affecting citrus production worldwide [1]. The pathogen enters host plant tissues through stomatal openings or wounds and then colonizes the apoplast causing the break of the epidermis due to cell hyperplasia. The infection manifests itself as erupted lesions on fruit, foliage and young stems. Severe disease can cause defoliation, dieback and fruit drop thereby reducing production volume, increasing prices and causing high market losses [1].

To better understand the molecular mechanisms underlying disease development, we previously focused on the identification and characterization of several bacterial components involved in Xcc virulence such as type III and type V protein secretion systems [2], [3], the exopolysaccharide xanthan [4] and a Xcc plant natriuretic peptide-like protein (XacPNP) [5]. The latter, XacPNP, shares significant sequence similarity and identical domain organization with plant natriuretic peptides (PNPs) but does not appear to have homologues in other bacteria [6]. We have recently demonstrated that recombinant XacPNP, much like its *Arabidopsis thaliana* homolog AtPNP-A, can cause plant physiological responses such as stomatal opening [5]. We also observed that the expression of *XacPNP* is induced upon infection and that lesions produced in leaves infected with a XacPNP deletion mutant were more necrotic than those observed with the wild type and the highly necrotic tissue led to earlier bacterial cell death in the mutant. This suggests that the plant-like bacterial PNP enables the plant pathogen to modify host responses in order to create conditions favorable to its own survival [5], [7].

To further the understanding of the citrus responses to Xcc, we performed a proteomic study of plant leaves proteins induced after the infection with this pathogen. Although there are studies describing global gene expression analysis on citrus plants during canker development that provide valuable information on the changes in gene expression [8], this is the first study identifying proteins whose abundance is modified by citrus canker. Among the differentially expressed proteins, we found candidates predicted to be involved in photosynthesis, carbon metabolism, biotic and abiotic stress responses. The roles of these proteins are discussed within the context of a compatible host-pathogen interaction. Moreover, to focus on the functional role of XacPNP in bacterial pathogenicity, we compared the proteomes of healthy plants and plants exposed to Xcc wild type as well as ΔXacPNP and analyzed host responses differentially triggered by these bacteria. We also assessed XacPNP-dependent physiological changes in the host in order to evaluate the role of the bacterial peptide in photosynthetic efficiency as well as the expression patterns of defense-related genes with a view to further elucidate the role of this protein in homeostatic changes of the host tissue. Finally, we discuss the effect of XacPNP on the primary metabolism of the host plant and the consequences on the arms race between the pathogen and the host.

## Results and Discussion

### Proteome Changes in Citrus Sinensis Leaves in Response to *X. citri* pv. *citri* Infection

**Figure 1:**
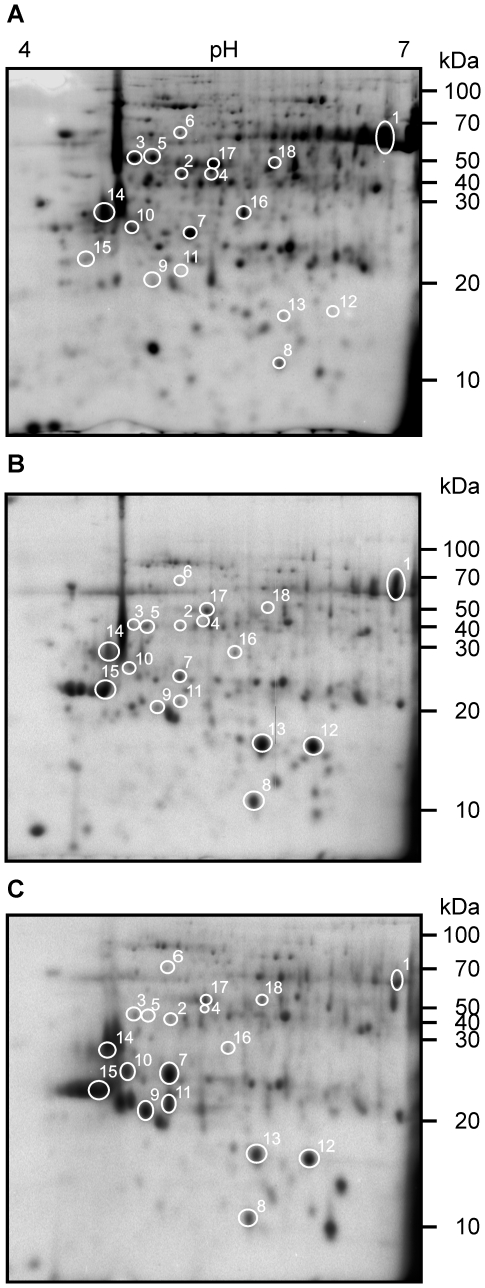
Protein profiles in 2-DE of total soluble proteins of Xcc-infected citrus leaves. Equal amounts of urea-buffer extracted proteins (200 µg) were separated on 7 cm pI 4–7 linear gradient strips in the first dimension and on 12% SDS-PAGE in the second dimension and stained with Coomassie blue. Proteins with significantly different expression levels between control and infected plants were marked with circles and numbered. Numbers refer to protein spot numbers on Table 1. Numbers on the right indicate molecular mass in kilodalton (kDa). (A) Citrus leaves infiltrated with Tris solution as control. (B) Citrus leaves inoculated with *X. citri* pv. *citri* 3 dpi and (C) 6 dpi.

Proteomics is a powerful technique to study changes in protein abundance and it is important in the identification of proteins directly involved in a particular biological process. For this reason we have applied it to discover citrus proteins that are directly affected following inoculation with Xcc. Total soluble proteins of whole leaves of *Citrus sinensis* 3 days post inoculation (dpi) with Xcc, the time at which the symptoms begin to appear, and at 6 dpi when the disease is fully established were separated using two-dimensional gel electrophoresis (2-DE) and representative images are shown (Figure 1). Protein extractions were performed from three independent leaves from three different plants at each time of infection and then characterized by 2-DE. Replica gels for each time point were compared with gels of proteins extracted from control leaves mock inoculated with 50 mM Tris-HCl pH 8. Comparative image analysis of the protein patterns indicated a number of spots, which were up- or down-regulated after pathogen challenge. Several spots demonstrated significant differences in intensities in samples from the Xcc inoculated leaves at 3 and 6 dpi respectively compared to the control (Figure 1). These spots were excised for identification by mass spectrometry and the proteins that could be identified are presented in Table 1. The results show that among the proteins differentially expressed during citrus canker development, proteins with a role in carbon metabolism, photosynthesis and stress defense responses are prominent. It is worth mentioning that these correspond well with transcripts induced in response to citrus canker as reported in a recent microarray global gene expression analysis [8].

**Table 1:** Proteins identified by 2-DE and MALDI-TOF in citrus leaves.

Spot	Accession	Protein name	Predicted MW/pI	MOWSE Score	Expect	Queries Matched	Fold change with XccWT	Fold change with ΔXacPNP
1	YP_740483	Rubisco large subunit (*C. sinensis*)	52388/6.29	129	4.4e-06	11	5.71±1.42 ↓	8.18±1.64 ↓
2	ABX84141	Rubisco activase (*Ipomoea batatas*)	48408/8.16	71	0.026	9	1.35±0.21 ↓	2.40±0.19 ↓
3	S39551	Rubisco activase Malus×domestica	48046/8.20	75	0.0039	10	2.06±0.14 ↓	2.63±0.49 ↓
4	S25484	Rubisco activase (fragment) (*Nicotiana tabacum*)	25913/5.01	70	0.014	6	ND^a^	ND
5	Q308Y6	Rubisco activase (*Gossypium hirsutum*)	46659/4.84	105	0.0001	11	ND	ND
6	YP_740460	ATP synthase CF1 alpha subunit (*C. sinensis*)	55452/5.09	138	5.4e-09	14	1.52±0.34 ↓	ND
7	Q2YGQ6	NADH dehydrogenase subunit F (fragment) (*Banara nitida*)	30842/9.58	73	0.0064	5	1.87±0.28 ↑	1.57±0.16 ↑
8	T10450	Superoxide dismutase (fragment) (*C. sinensis*)	12777/5.82	66	0.037	4	2.05±0.62 ↑	2.51±0.37 ↑
9	Q8LKR8	NADP-dependent sorbitol-6-phosphate dehydrogenase (fragment) (*Prunus emarginata*)	28355/9.16	74	0.0055	6	5.68±1.14 ↑	5.87±0.97 ↑
10	T09831	Alcohol dehydrogenase (fragment) (*G. hirsutum*)	23770/6.58	65	0.037	5	3.78±0.84 ↑	3.99±0.91 ↑
11	Q6Q4B3	PR10-5-like protein (*G. barbadense*)	17172/5.21	68	0.019	7	22.14±3.84 ↑	19.38±1.97 ↑
12	ABK06393	Stress-related protein PR10 - Polyketide-cyc2 (*C. sinensis*)	17593/5.67	70	0.033	5	21.00±5.12 ↑	24.82±4.03 ↑
13	Q9M3Z4	bZip transcription factor (*Cicer arietinum*)	37012/8.38	76	0.0029	9	10.68±1.98 ↑	12.52±1.38 ↑
14	Q52Z99	6-4 photolyase (*Dunaliella salina*)	67081/9.51	74	0.037	12	1.25±0.39 ↑	1.12±0.28 ↑
15	T04362	GTP-binding protein yptm3 (*Zea mays*)	23042/6.96	70	0.009	7	17.01±2.97 ↑	19.03±2.71 ↑
16	AAG52805	Ribosomal protein 4 (*Leptobryum wilsonii*)	21999/10.19	78	0.006	7	3.02±0.29 ↓	2.870±0.36 ↓
17	Q2KLP5	Small ribosomal protein subunit 4 (fragment) (*Gyroweisia tenuis*)	22399/10.82	69	0.016	7	1.22±0.21 ↓	2.01±0.15 ↓
18	Q6Y682	38 kDa ribosome-associated protein (*Chlamydomonas reinhardtii*)	44711/9.45	67	0.025	12	2.20±0.42 ↓	2.24±0.36 ↓

Summary of the differentially expressed proteins identified from citrus leaf tissue infected with Xcc wild type and ΔXacPNP Fold change of protein levels relative to mock infiltrated leaves were quantified and the results for 6 dpi are shown.

a ND: not detected.

### Proteins Related to Carbon Metabolism and Photosynthesis

Among the proteins with a role in carbon metabolism, we noted the large subunit of Ribulose-biphosphate carboxylase (Rubisco) and Rubisco activase. Rubisco is the key enzyme in the Calvin cycle and catalyzes carbon dioxide fixation while Rubisco activase regulates Rubisco activity by hydrolysing ATP to promote the dissociation of inhibitory sugar phosphates [9]. The levels of both proteins decreased at 3 and 6 dpi compared to the control (Figure 1, spots 1–5). In all the replicates, the differences were more pronounced at 6 dpi, when the disease phenotype was observed, than at 3 dpi (Figure 1C). The proteomes also showed significant reduction in the level of the α subunit of chloroplast F1 ATP synthase (Figure 1, spot 6). The decreases observed in the levels of these three proteins are consistent with impaired photosynthetic efficiency in infected leaves. We also noted an induction of the expression of the NADH dehydrogenase (Ndh) subunit F (Figure 1, spot 7). The Ndh complex is localized in the chloroplast and its main function is to act as an intermediate in chlororespiration and plants which lack a functional PSII show a 10-fold relative increase in the Ndh complex and the plastid terminal oxidase [10]. These data indicate that our experimental conditions the induction of the Ndh complex is likely to be diagnostic for a dysfunction of photosynthesis.

It is well documented that in plant-pathogen interactions, pathogens aim to overcome host defense responses while plants employ a battery of responses to limit pathogen growth and thus disease. In this “arms race” plants induce an array of defense mechanisms that although not always successful, demand a substantial reallocation of energy towards the defense response and this provision of energy for defense processes necessitate the modification of primary metabolism [11]. Our results are indicative of a decrease in photosynthesis during citrus canker infection. The decrease in the expression of sugar-regulated photosynthetic genes such as Rubisco and Rubisco activase and also of ATP synthase and the increase in NADH dehydrogenase support the case for a reduction in photosynthetic efficiency. Incidentally, similar observations were made in plants infected with biotrophic fungal pathogens that also reduced photosynthetic rates [12]. This down-regulation has been preceded by an accumulation of hexoses, probably due to an increase in invertase activity triggering signaling pathway that represses photosynthetic gene expression [12], [13]. Our results are also in agreement with whole transcriptome data from citrus canker that revealed a down-regulation of Rubisco and Rubisco activase among other photosynthetic genes, as well as an induction of invertase expression and thus being consistent with the hypothesis of “sugar sensing” [8].

### Identification of Antioxidant Proteins

A protein induced at 3 and 6 dpi (Figure 1, spot 8) was identified as superoxide dismutase (SOD). The overproduction of reactive oxygen species (ROS) in plant cells under stress conditions, such as pathogen infection, is a strategy generally used by plants to limit pathogen growth [14]. However, when ROS levels are above a threshold level, deleterious effects such as necrosis of plant cells may occur. Therefore, excess ROS are scavenged by enzymes and redox metabolites [15]. Superoxide dismutases constitute the first line of defense against ROS, enzymes that catalyse the dismutation of ROS such as O_2_ to H_2_O_2_ and thereby ameliorate their toxic effects. Based on a model proposed by Casano et al. (2000), the Ndh complex also plays a protective role in photosynthesis under oxidative stress since it consumes ROS produced in the chloroplast under most stress conditions [16].

### Proteins Related to Abiotic and Biotic Stress Responses

Among the proteins that showed augmented levels after Xcc challenge we observed the presence of a NADP-dependent sorbitol-6-phosphate dehydrogenase (S6PDH) (Figure 1, spot 9), a key enzyme in sorbitol synthesis. Accumulation of sorbitol in plants changes in response to environmental conditions and thus enables the regulation of their osmotic potential and therefore adapt to salt or drought stress. A correlation between sorbitol-over-production and higher levels of defense-related proteins was also observed [17]. It is likely that in our system higher levels of S6PDH after Xcc infection mark a response to the osmotic stress caused by the pathogen.

Another protein that showed increased levels was identified as alcohol dehydrogenase (Figure 1, spot 10), a class of enzymes that are involved cell-wall synthesis and remodelling. Induction of a number of genes encoding different types of alcohol dehydrogenases has been shown to be related to plant defense responses. These include genes for cinnamyl alcohol dehydrogenases (CADs) [18], and aromatic alcohol dehydrogenases such as ELI-3 from Arabidopsis and parsley [19] as well as a novel potato gene, *drd-1* (defense-related alcohol dehydrogenase 1) [20].

Two highly induced proteins corresponded to the family of pathogenesis related (PR) proteins (Figure 1, spots 11 and 12), the first being homologous to the PR-10-5 and the second to a citrus stress-related protein that has high homology also to PR10 [21]. PRs are defined as proteins encoded by the host plant induced specifically when challenged with a pathogen [22]. PR proteins do not only accumulate locally in the infected leaf, but are induced systemically and associated with the development of systemic acquired resistance (SAR) against further infection by fungi, bacteria and viruses. The PR-10 family is structurally related to ribonucleases and although it is tempting to propose that these intracellular PR proteins may be active against viruses their capacity to cleave viral RNA remains to be demonstrated [23].

### Proteins Related to the UV-Response

The induced spot 13 (Figure 1) is a protein which is homologous to a hypothetical protein of *Cicer arietium* (Table 1). BLAST analysis showed that this protein may be a basic region/leucine zipper (bZIP) transcription factor showing high (55% identity and 68% similarity) homology to bZIP56. Genetic and molecular studies on *A. thaliana* bZIP (AtbZIP) factors show that they regulate diverse biological processes such as pathogen defense, light and stress signaling, seed maturation and flower development [24]. AtbZIP56 (also called LONG HYPOCOTYL5, HY5) is involved in promoting photomorphogenesis by regulating the expression of several light-inducible genes. It was also observed that HY5 directly regulates the transcription of photosynthesis-related genes during seedling photomorphogenic development [25]. Given that the photosynthetic machinery is likely to be injured in citrus leaves infected with Xcc, HY5 over-expression may be a response that can contribute to at least a partial repair of the damage.

The induced spot 14 (Figure 1) corresponds to the photolyase enzyme UVR-3 required for photorepair of 6-4 photoproducts in *A. thaliana* [26]. Plant absorption of UV-B radiation induces the formation of two classes of pyrimidine dimers in DNA (CPDs) that account for the majority in these lesions with the remainder being mainly pyrimidine (6-4) and pyrimidone dimers (6-4PPs) [27]. Since the accumulation of CPDs and 6-4PPs in DNA must be prevented if cell viability is to be maintained, higher plants have evolved at least two major mechanisms for their removal. The dark repair pathway occurs in the absence of visible light and involves several enzyme-mediated steps consisting of base and nucleotide excision repair mechanisms. The light-dependent pathway is mediated by photolyase enzymes and is the major pathway for the removal of CPDs and 6-4PPs lesions in higher plants [27]. In addition to these repair mechanisms, plants have several photoprotective pigments such as flavonoids and carotenoids. We have observed that citrus leaves infected with Xcc wild type have lower carotenoids levels (see below), suggesting that the induction of proteins involved in repair mechanism during citrus canker is aimed at counteracting the lack of photoprotection. Since HY5 triggers the transcriptional induction of several UV-B-responsive genes [25] and we observed higher levels of this transcription factor in Xcc infected leaves, we may consider that the higher expression of UVR-3 could be an effect of increased levels of HY5 during the infection.

Recent research has expanded our knowledge of plant defense mechanisms. To optimally fulfil the many different defense requirements, several signaling pathways are likely to overlap and interact to produce a comprehensive and integrated response. Even if there is specificity in stress signal perception, the signaling pathways and the final responses may appear somewhat unspecific. As a point in case, it is well known that UV-B radiation acts through signaling pathways whose components closely resemble those for pathogen resistance [28]. Interestingly, we found a hypothetical protein resembling a transcription factor implicated in the UV-B stress response. UV stimulates transcription of genes important for defense and in addition the UV treatment of *A. thaliana* plants before the inoculation of *Hyaloperonospora parasitica* induces a dose-dependent resistance to the pathogen [29]. These results link UV-induced DNA repair systems to the activation of the immune response. Others tested signaling pathways induced by oligosaccharide elicitors (OEs), systemin and UV-B radiation and found that pretreatment with systemin and OEs transiently reduced the MAPK response to a subsequent treatment with the same or a different elicitor [30]. In contrast, MAPK activity in response to UV-B increased after pretreatment with systemin and OEs. These experiments provide evidence for the presence of signaling components that are shared by systemin, OEs, and UV-B [30]. It is well known that UV-B responses are mediated by nonspecific signaling pathways that involve DNA damage, ROS and wound/defense signaling [31]. Since citrus plants exposed to Xcc were grown in absence of UV radiation, the observed induction of two proteins involved in UV-responses may be attributed to a direct effect of pathogen infection. Interesting, to our knowledge this is the first report inferring that plant defense responses to a bacterial pathogen may trigger a UV-like response. Further studies will clarify the commonalities of the responses to UV radiation and pathogen stress.

### Proteins Related to Plant Protein Trafficking

The highly induced spot 15 (Figure 1) is a protein with significant homology to At-Rab2. The Rab family of small GTPases contributes to the specification of membrane identity, the accuracy of vesicle targeting and the recruitment of molecular motors to membranes [32]. Rab2 subfamily is localized on cis-Golgi membranes and interacts with Golgi matrix proteins. Rab2 is also implicated in the maturation of vesicular tubular clusters (VTCs), which are microtubule-associated intermediates in transport between the ER and Golgi apparatus. In plants, Rab2 regulates vesicle trafficking between the ER and the Golgi bodies and is expressed in maturing pollen and rapidly growing organs of germinating seedlings [33]. One of the first reactions observed in cells under pathogen attack is the reorganization and polarization of the cytoskeleton in the site of attack to accomplish a rapid transport of antimicrobial compounds, proteins and cell-wall material to the plant-pathogen interface [34]. Here we propose that Rab2 induction in response to Xcc infection is part of specific and increased trafficking that occurs as part of the defense response.

### Proteins Related to Plant Chloroplast Ribosomal Proteins

The spots 16 to 18 (Figure 1) are homologues of chloroplast ribosomal proteins and are down-regulated as a consequence of infection. Chloroplasts have been identified as organellar targets of bacterial effector proteins and some of these effectors possess transit peptides resembling chloroplast-targeted proteins [35]. As an example it has recently been demonstrated that the *Pseudomonas syringae* effector HopI1 is targeted to the chloroplast and induces remodeling of the thylakoid structure, affecting chloroplast function and promoting bacterial virulence by suppressing plant defenses, implying an active function of these organelles in the defense response [36]. In this context we may propose that Xcc is directly or indirectly modifying chloroplast structure that in turn contributes to suppressing plant cell death via its effectors and that chloroplastic ribosomal proteins are be targets for plastid rearrangements.

### XacPNP Prevents Reduction of Host Photosynthetic Proteins

**Figure 2:**
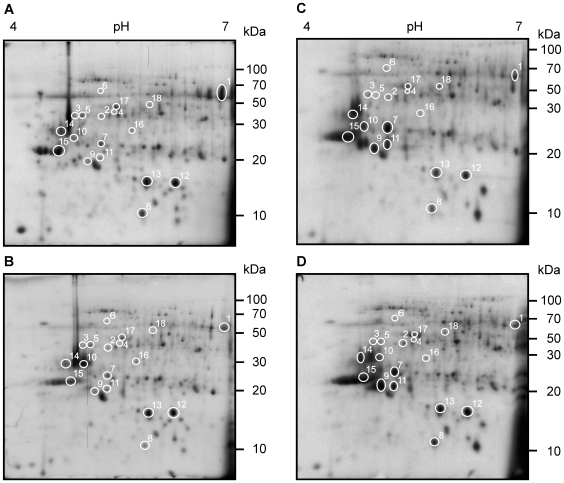
Protein profiles in 2-DE of total soluble proteins of Xcc and ΔXacPNP-infected citrus leaves. Equal amounts of urea-buffer extracted proteins (200 µg) were separated on 7 cm pI 4–7 linear gradient strips in the first dimension and on 12% SDS-PAGE in the second dimension and stained with Coomassie blue. Spots numbers are the same as in Figure 1. Numbers on the right indicate molecular mass in kilodalton (kDa). (A) Citrus leaves inoculated with Xcc wild type (XccWT) 3 dpi and (C) 6 dpi and (B) with ΔXacPNP 3 dpi and (D) 6 dpi.

A proteomic analysis was also performed on leaves infected with ΔXacPNP strain. The ΔXacPNP lesion phenotype displays larger necrotic areas indicative of a general deterioration of the condition of the infected tissue [5]. Figure 2 shows the principal differences between host leaves proteins inoculated with wild type (Figure 2A) and ΔXacPNP (Figure 2B) at 3 dpi as well as at 6 dpi (Figure 2C and 2D), respectively. Compared with control leaves, leaves infected with the ΔXacPNP strain showed an 8-fold decrease in Rubisco levels at 3 dpi whereas leaves infected with the wild type strain showed only a 2-fold decrease (Figure 2A and 2B, spot 1). These significant differences (p<0.05) according to ANOVA became less pronounced at 6 dpi when leaves infected with ΔXacPNP maintained their Rubisco levels while wild type infected leaves showed a decrease of almost 6-fold (Figure 2C and 2D, spot 1, Table 1). This is consistent with the increased necrotic tissue observed earlier in leaves infiltrated with the mutant strain in which plant primary metabolism appeared to have slowed down. In addition, Rubisco activase and ATP synthase proteins were also slightly lowered in ΔXacPNP infection (Figure 2, spots 2–6) as well as plastid ribosomal proteins (Figure 2, spots 16–18, Table 1). It turns out that the main differences between infections with the two bacterial strains are in proteins with a key role in carbon metabolism and photosynthesis. In contrast, no differences were observed in the levels of typical defense proteins (Figure 2, spots 7–15, Table 1). In particular, no differential induction of proteins involved in the oxidative stress response was observed and the same holds true for pathogenesis related proteins at 6 dpi (Figure 2, spots 8, 11 and 12, Table 1), suggesting a similar progression of the defense program in tissues infiltrated with both strains.

### Photosynthetic Parameters and Pigment Content of Orange Leaves Infected with Xcc Wild Type and ΔXacPNP

**Figure 3:**
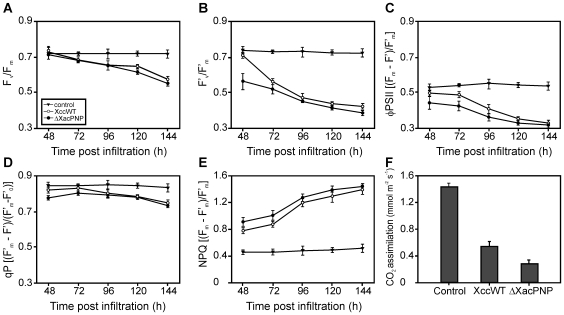
Photosynthetic parameters measurements. (A) Potential quantum efficiency of PSII (*F*_v_/*F*_m_) of control (filled triangles), XccWT (open circles) and ΔXacPNP (solid circles) infiltrated citrus leaves (B) Effective quantum efficiency of PSII (*F*′_v_/*F*′_m_) (C) PSII operating efficiency (φ_PSII_). (D) Photochemical fluorescence quenching (qP). (E) Nonphotochemical fluorescence quenching (NPQ). B–E were measured in control, XccWT and ΔXacPNP infiltrated citrus leaves as represented as in A. (F) CO_2_ assimilation measured on infiltrated citrus leaves at 48 hpi. The results are the mean of five replicates and error bars represent the standard deviations.

Given the link between bacterial infection and the decrease in proteins involved in photosynthesis, we analyzed photosynthetic efficiency by measuring chlorophyll fluorescence. This is a powerful technique to monitor photosynthetic performance even before visible signs of stress appear [37]. Chlorophyll fluorescence parameters were examined in leaves infiltrated with wild type and ΔXacPNP strains infiltrating both strains at 10^7^ CFU/ml and after 48, 72, 96, 120 and 144 hours. Figure 3A shows reduced photosynthetic efficiency in wild type infiltrations as compared to control leaves and interestingly, the reduction is bigger in ΔXacPNP infiltrated tissue. In addition, we also noted lower values of maximum quantum efficiency of photosystem II (PSII) (F_v_/F_m_) in dark-adapted leaves (Figure 3a), lower potential quantum efficiency of PSII at 100 µmol quanta m^−2^ s^−1^ (Figure 3B), lower PSII operating efficiency (φ_PSII_) (Figure 3C) and lower photochemical quenching (qP) (Figure 3D) in infiltrated leaves as compared to the controls. It was therefore concluded that bacterial infection diminishes maximum quantum efficiency of PSII. In the case of non-photochemical quenching higher values were observed for infiltrated leaves (Figure 3E), suggesting a higher heat loss in the infected tissues. The physiological data are therefore consistent with the results of the proteomic analyses and the phenotype of ΔXacPNP infected tissue that showed increased impairment compared to tissue infected with wild type bacteria [5]. Further, we measured CO_2_ assimilation at 48 hpi and observed a decrease of the assimilation rate of more than 60% for Xcc while for ΔXacPNP this value declined to 80% of the control (Figure 3F). In summary, photosynthetic performance decreases in infected leaves corroborating the proteomic assay in the down-regulation of photosynthesis related proteins and the decrease is markedly more dramatic after infection with ΔXacPNP. The results thus shed light on the role of photosynthetic rates in the plant-pathogen interaction (reviewed in [11]). Several studies on photosynthesis in compatible interactions have shown that rates of photosynthesis are reduced after treatment with pathogens [12], [38]–[40]. In that context and since we determined that ΔXacPNP dies earlier than the wild type bacteria in orange leaves [5] we suggest that XacPNP is maintaining host tissue in better conditions for biotrophic pathogen survival and that this tissue status is related to a counteracting of the pathogen-induced decrease in photosynthesis. We have also analyzed chlorophyll a and b and carotenoids content in infected leaves (Table 2). As expected, infection reduces chlorophylls and carotenoids content, though the infection with ΔXacPNP yields lower levels of pigments compared to Xcc infection, lending further support to the idea that the presence of XacPNP offsets the detrimental effect of the pathogen on the photosynthetic apparatus.

**Table 2:** Pigments content of infiltrated citrus leaves.

Treatment	3 dpi			6 dpi		
	Chl a	Chl b	Carotenoids	Chl a	Chl b	Carotenoids
Mock	29.15±1.80	12.60±0.71	6.29±0.37	28.23±1.93	11.62±1.57	6.56±0.66
XccWT	20.76±1.53	11.34±1.18	6.28±0.75	7.37±0.64	5.03±0.57	4.60±0.48
ΔXacPNP	19.52±1.38	10.38±0.48	6.36±0.57	4.62±0.39	3.31±0.36	2.85±0.35

Pigments were determined in mock and infected citrus leaves at 3 and 6 dpi as described by [45]. Mock infiltrations were performed with 10 mM MgCl_2_.Values indicate the mean of three experiments and are expressed in µg/cm^2^ of tissue.

### XacPNP Is Not Involved in Innate Immunity, the Stress Oxidative Response or the Induction of Pathogenesis-Related Proteins

**Figure 4:**
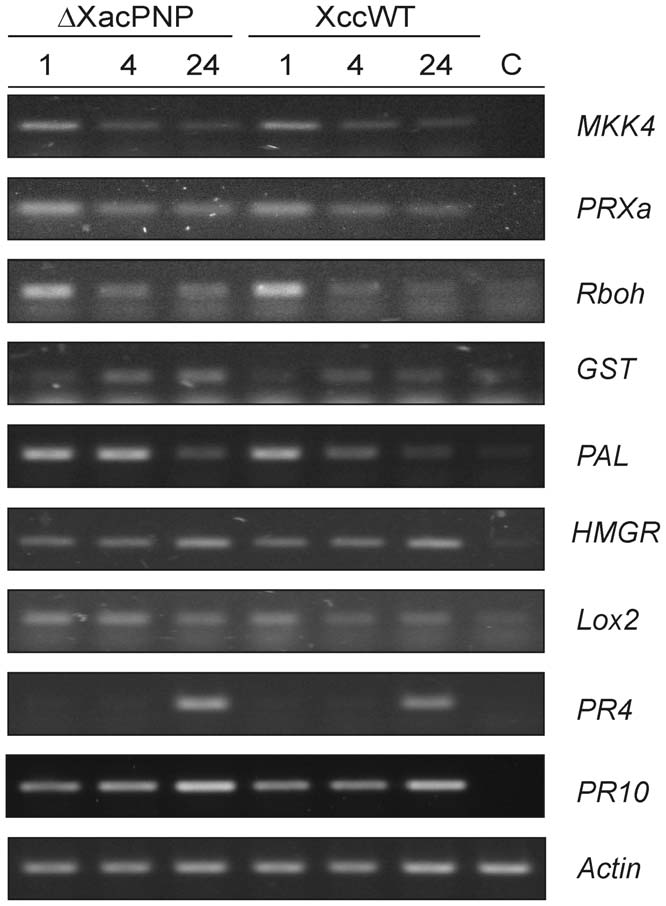
Expression of defense-related genes in citrus leaves infected with ΔXacPNP and Xcc (XccWT). RT-PCR were performed in RNA samples taken at 1, 4 and 24 hpi. MKK4, MAP kinase kinase 4; PRXa, peroxidorexin; Rboh, NADPH oxidase; GST, glutathione-S-transferase; PAL, phenylalanine ammonia lyase; HMGR, 3-hydroxy-methylglutaryl CoA reductase; Lox2, lipoxygenase 2. As controls (C) leaves infiltrated with 10 mM MgCl_2_ at the times stated were analyzed. While in the figure the control at 1 hpi is shown, similar results were obtained for 4 and 24 hpi.

The proteomics results indicate that the ΔXacPNP strain is inducing similar plant defense proteins to an equivalent amount than the wild type including PR10 protein, alcohol dehydrogenase and NADP-dependent sorbitol-6-phosphate dehydrogenase. In order to confirm that XacPNP is not directly engaged modulating defense or resistance responses in plants, we performed an expression analysis at 1, 4 and 24 hpi by RT-PCR of genes diagnostic for in the innate immunity, oxidative stress and defense responses in citrus leaves infiltrated with either Xcc or ΔXacPNP (Figure 4) and observed similar levels and patterns of induction for all of them in both strains. In particular, transient expression of MAP kinase kinase 4 (MKK4) peaking at 1 hpi was observed indicating the onset of an innate immune response in citrus leaves regardless of the strain used. The oxidative burst associated proteins peroxidorexin (PrxA), NADPH oxidase (RbohB) were also elicited 1 hpi while glutathione-S-transferase (GST) showed a slight induction 4 hpi. In the case of proteins related to secondary metabolite formation phenylalanine ammonia lyase (PAL) was induced 1 hpi and 3-hydroxy-methylglutaryl CoA reductase (HMGR) at 24 hpi. Finally, the defense-related proteins lipoxygenase 2 (Lox2) showed no induction with any of the two strains analyzed at the times stated while the pathogenesis related proteins PR4 and PR10 showed an induction at 24 hpi, being consistent with the proteomic results in which we observed the presence of PR10 in both strains at both times. Since no difference in the induction of pathogen response genes occurred, we propose that the primary function of XacPNP is to sustain host photosynthesis and consequently homeostasis for the benefit of the pathogen [5].

### *X. citri* pv. *citri* Uses a Natriuretic Peptide to Improve Plant Photosynthesis during Infection

**Figure 5:**
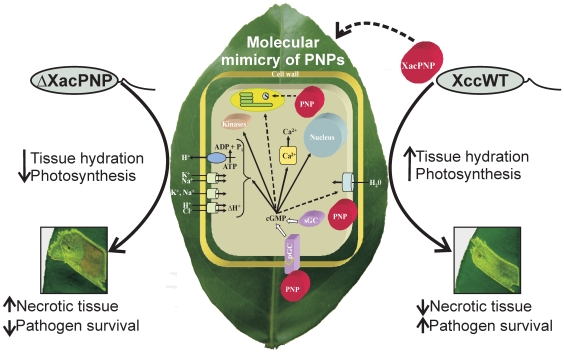
Model of XacPNP action in citrus canker. Typical citrus leaves lesions with ΔXacPNP (left) and Xcc wild type (right) are observed in the bottom images where differences in tissue necrosis are shown. On the leaf is a proposed model for PNP-dependent cellular processes (described in the text). Solid arrows indicate experimentally confirmed functions and dashed arrows indicate proposed mechanisms.

The relationship between photosynthesis and its particular role in energy generation for the defense program has been a matter of controversy. Although it is rational to consider that energy and carbon skeletons required for plant defense has to be sustained by increases in photosynthesis, several studies demonstrate that this is not the case (reviewed in [11]). It has been proposed that a priority in infected plants is the production of defense-related compounds and that the disposable cellular activities such as the primary metabolism may be reduced [11]. In fact, many reports on photosynthesis and plant defense have shown that rates of photosynthesis are reduced after treatment with virulent or avirulent pathogens with the main difference being the timing of this reduction [12], [38]–[40]. *X. citri* pv. *citri* is a Gram-negative bacterial pathogen that uses the type III protein secretion system to translocate effector proteins into the host cell [2]. These effectors suppress basal defense responses and collectively contribute to the disease. Many effector proteins are believed to be localized in the host cell cytoplasm or in the nucleus [41]. In spite of the vast number of pathogen effector proteins identified to-date the *P. syringae* effector HopI1 is the only one that seems targeted to the chloroplast where it induces remodeling of the thylakoid structure [42]. Chloroplast function can also be altered by the fungal virulence protein, ToxA produced by the wheat pathogen *Pyrenophora triticirepentis* that interacts with a plastid protein necessary for thylakoid membrane organization [43]. We propose that Xcc has acquired and adapted a plant protein and mimics its function to maintain host conditions better suited to its biotrophic lifestyle and doing so by directly or indirectly modulating and/or sustaining chloroplast function. Accordingly, we suggest a model in which XacPNP produced by *X. citri* pv. *citri* mimics host PNP functions. These include the increase of cGMP either directly through receptor like guanylyl cyclase (particulate GCs) or indirectly through the activation of soluble guanylyl cylases (sGCs). Among the effects produced by PNP and/or cGMP-dependent signalling are the regulation of plant cell homeostasis and water uptake by modification of ion channels, activation of kinases and transcription, and the regulation of chloroplast function. Consequently, XacPNP enables more efficient photosynthesis resulting in a delay in tissue necrosis to the benefit of the biotrophic pathogen (Figure 5).

In summary, we propose that XacPNP overcomes host earlier necrosis by counteracting shutting down of photosynthesis and thus allows bacterial prolonged survival on the host. To our knowledge, this is the first report in which primary metabolism is manipulated by a plant-like molecule operating in a pathogen.

## Materials and Methods

### Plant Material and Plant Inoculations

Orange plants (*Citrus sinensis* cv. Valencia) were grown in a green house at 25°C with a photoperiod of 16/8 h. Xcc wild type and ΔXacPNP strains were grown at 28°C in Silva Buddenhagen (SB) [5] to OD_600_ of 1, harvested by centrifugation, and resuspended in 10 mM MgCl_2_ or 50 mM Tris-HCl pH 8 at a density of 10^7^ cfu/ml. Bacterial infiltrations into leaves were performed with needleless syringes.

### Plant Treatment and Protein Extraction

After 48 hpi (hours post infiltration), citrus leaves protein extracts were prepared by pulverization of leaves under liquid nitrogen followed by re-suspension in 50 mM Hepes-KOH buffer pH 7.5, 330 mM sorbitol, 5 mM sodium ascorbate, 2 mM EDTA, 1 mM MgCl2, 1 mM MnCl2 and 0.33 mM PMSF in a ratio 1∶2 (w/v). The samples were centrifuged at 12000×g at 4°C, for 20 min and the supernatants precipitated with 10% trichloroacetic acid (TCA) in acetone. Precipitated proteins were collected by centrifugation at 13400×g for 10 min at 4°C. The pellet was washed 3 times with ice-cold 80% acetone, air dried at room temperature and resuspended in urea buffer (9 M urea, 2 M thiourea and 4% 3-[(3- Cholamidopropyl)dimethylammonio]-1-propanesulfonate (CHAPS)] for at least 1 h with vigorous vortexing at room temperature. The concentration of all protein extracts was estimated using a modified Bradford assay [44]. Briefly, 5 µL of each protein sample and BSA standards were mixed with 20% (v/v) Bradford reagent (Bio-Rad, Hercules, CA, USA) containing 0.1 mm hydrochloric acid in a final volume of 1 mL. The absorbance (at 595 nm) of each sample mixture, which is proportional to the quantity of solubilized protein, was measured using a Genesis 5 Spectrophotometer (Milton Roy, Groton, CT, USA).

### Two-Dimensional (2-DE) Gel Electrophoresis

Soluble proteins (200 µg) were mixed with 0.8% (w/v) dithiothreitol (DTT), 0.2% (v/v) ampholytes pH 3–10 (BIO-RAD, Hercules, CA), 0.002% bromophenol blue and the volume was adjusted to 125 µL using urea buffer. The samples were then used to passively rehydrate linear 7 cm IPG strips, pH 4–7 (BIO-RAD) overnight at room temperature. The strips were subjected to isoelectric focusing (IEF) using the Ettan™ IPGphor II™ (GE Healthcare, Amersham, UK), in a step wise programme for a total of 3,700 Vhrs at 20°C. Prior to the second dimension, the strips were equilibrated twice for 10 min with gentle shaking in an equilibration buffer (6 m urea, 2% (w/v) SDS, 0.05 m Tris-HCl, pH 8.8 and 20% (v/v) glycerol) firstly containing 1% (w/v) DTT and then 2.5% (w/v) iodoacetamide. The strips were then loaded to 12% SDS-PAGE gels and electrophoresed at 120 V until the bromophenol blue dye reached the bottom of the gels. The gels were stained with Coomassie Brilliant Blue, imaged with the PharosFX™ plus molecular imager scanner (BIO-RAD) and analysed using the PD-Quest software (BIO-RAD). Spots that showed reproducible differences in all three gels for each analyzed time as determined by the T-test from PD-Quest (p<0.05) were selected for mass spectrometry analysis.

### In-Gel Trypsin Digestion and Mass Determination

Spots of interest were excised manually and transferred into sterile microcentrifuge tubes. The gel pieces were washed twice with 50 mM ammonium bicarbonate for 5 min each time and a third time for 30 min, vortexing occasionally. The gel pieces were then destained two times with 50% (v/v) 50 mM ammonium bicarbonate and 50% (v/v) acetonitrile for 30 min, vortexing occasionally. The gel pieces were dehydrated with 100 µL (v/v) acetonitrile for 5 min, and then completely desiccated using the Speed Vac SC100 (ThermoSavant, Waltham, MA, USA). Proteins were in-gel digested with approximately 120 ng sequencing grade modified trypsin (Promega, Madison, WI, USA) dissolved in 25 mM ammonium bicarbonate overnight at 37°C. The protein digestion was stopped by adding 50–100 µL of 1% (v/v) trifluoroacetic acid (TFA) and incubating 2–4 h at room temperature before storage at 4°C until further analysis.

Prior identification, the samples were cleaned-up by reverse phase chromatography using ZipTip _C18_® (Millipore, Billerica, MA, USA) pre-equilibrated first in 100% (v/v) acetonitrile and then in 0.1% (v/v) TFA and eluted out with 50% (v/v) acetonitrile. One µL from each sample was mixed with the same volume of α-cyna-hydroxy-cinnamic acid (CHCA) matrix and spotted onto a MALDI target plate for analysis using a MALDI-TOF mass spectrometer, the Voyager DE Pro Biospectrometry workstation (Applied Biosystems, Forster City, CA, USA) to generate a peptide mass fingerprint. All MALDI spectra were calibrated using sequazyme calibration mixture II containing angiotensin I, ACTH (1–17 clip), ACTH (18–39 clip), ACTH (7–38 clip) and bovine insulin (Applied Biosystems). The NCBI and MSDB peptide mass databases were searched using MASCOT (http://www.matrixscience.com/search_form_select.html) with 100 ppm accuracy and oxidation as variable modification selected.

### Chlorophyll Fluorescence Determinations and CO_2_ Assimilation

Chlorophyll fluorescence parameters were measured using a portable pulse amplitude modulation fluorometer (Qubit systems Inc., Ontario, Canada) connected to a notebook computer with data acquisition software (Logger Pro3 Version). The minimal fluorescence level (F_0_) in the dark-adapted state was measured when only the LED light was turned on. The output from the LED light is insufficient to drive photosynthesis and does not disturb the dark-adapted state. The maximal fluorescence level in the dark-adapted state (F_m_) and the maximal fluorescence level during illumination (F_m_′) were measured by a 0.8s saturating pulse at 5000 µmol m^−2^ s^−1^. F_m_ was measured after 30 min of dark adaptation. F_m_′ was measured with actinic light source of photon flux density (PPFD) 100 µmol m^−2^ s^−1^. The minimal fluorescence level during illumination (F_0_′) was calculated from measured values of F_0_, F_m_, F_m_′ and F_v_ [37]. Photosynthetic parameters: potential (F_v_/F_m_), effective (F′_v_/F′_m_) quantum efficiency of PSII and PSII operating efficiency {φ_PSII_ = [(F′_m_−F′)/F′_m_]}, photochemical {qP = [(F′_m_−F′)/(F′_m_−F′_o_)]} and nonphotochemical {NPQ = [(F_m_−F′_m_)/F′_m_]} fluorescence quenching were calculated as described [37]. Gas exchange measurements were carried out using an open-gas portable photosynthesis system (Qubit systems Inc., Ontario, Canada), an infra red gas analyzer (IRGA) that measures the concentration of CO_2_ in a gas entering a leaf chamber, and the concentration of CO_2_ in the same gas after it leaves chamber. Measurement of this CO_2_ differential, and measurement of the flow rate of gas through the chamber, allows calculation of photosynthetic CO_2_ fixation rate. Measuring conditions were saturating light (1500 µmol photons m^−2^ s^−1^), ambient temperature (between 25°C–30°C), flow rate of 500 mL min^−1^ and CO_2_ concentration (400 ppm). Leaves from four different plants were measured for each treatment.

### Plant Pigments Determination

Discs of 1 cm^2^ were excised from orange leaves and placed into plastic tubes with 1 mL ethanol 100% (v/v), sealed and incubated in the dark at 60°C for 48 h. Absorbances of the clear extract at 470, 648, and 664 nm were recorded and concentrations of chlorophylls *a*, *b*, *a*+*b* and carotenoids were calculated as previously described [45].

### RNA Preparation and RT-PCR

RNA samples from citrus leaves were isolated using Trizol reagent (Invitrogen) according to the manufacturer's instructions. Samples (1 µg) were then subjected to reverse transcription using the M-MLV Reverse Transcriptase (Promega) and PCR reaction (25 cycles of 94°C (1 min), 58°C (1 min) and 72°C (2 min)) followed by incubation at 72°C for 5 using the following oligonucleotides: GGCACCCTCGATACTTTGTT and TAATTCCCTCCGTAGGCATC for MKK4; AGCCGCTCTCATTTCCTCTA and TTGATCGAAAACAGCCTCTG for PrxA; TCTAGGAAAATCAGGATTGTTATGTC and AAAGCCAGATAGATTCAGATACAAGA for RbohB; GTTCATCAGATATCTTAAGGCTGGTA and AACCTACTTGGAAACACACTAGAAGA for GST; CTTGAATTATCCATAGAGACACCAAT and ATAATGGAACATATCTTGGATGGTAG for PAL; AATATGTAGATCCTTCCCATCATTTA and AAACTAATGTGGCTACACTGGTAGAG for HMGR; GTATAAATATTGCCCAAAGTTCACAG and GCCTTAAAACAATGGGTTACTAACTA for Lox2; GTGTGATTCTGTCACTTTGTCTACTG and ACTGTTTGTGACCCTTAAGCAC for PR4; ACATGATCAATAGTAGGGATGTTAGC and AAAGTTGTTCAAACTTTTTGTCCTT for PR10; and ACGTGAATTCTAGTGTTTCGATAAGT and TCAATTGGATACTTCAAAGTCAAAAT for actin. PCR products were electrophoresed in a 1% agarose gel and photographed with FOTO/Analyst. Investigator Eclipse (Fotodyne) and Gel-Pro Analyzer Software 3.1 (Media Cybernetics) were used to measure the intensity of each band.
